# Supplementary material for: Seebeck and Figure of Merit Enhancement by Rare Earth Doping in Yb14-xRExZnSb11 (x = 0.5)
Source: Materials (Basel). 2019 Mar 3;12(5):731. doi: 10.3390/ma12050731 (PMC6427333; doi:10.3390/ma12050731)
Supplement: Supplementary file 1 [file materials-12-00731-s001.pdf]

Supplemental Information for

Seebeck and Figure of Merit Enhancement by Rare Earth Doping in $Yb_{14-x}RE_xZnSb_{11}$ (x = 0.5)

Elizabeth L. Kunz Wille[1], Navtej S. Grewal[1], Sabah Bux[2] and Susan M. Kauzlarich[1*]

[1] Department of Chemistry, University of California, Davis, One Shields Avenue, Davis, CA 95616
[2] Jet Propulsion Laboratory, California Institute of Technology, 4800 Oak Grove Drive, Pasadena, CA 91109
[*] Correspondence: smkauzlarich@ucdavis.edu

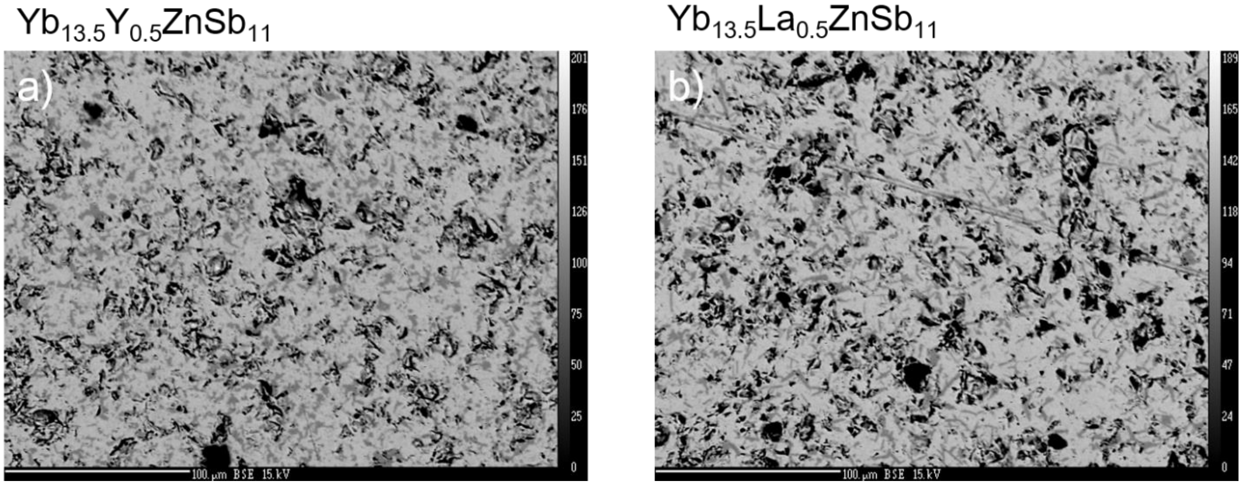

Figure S1. Microprobe backscatter electron images of (a) $Yb_{13.5}Y_{0.5}ZnSb_{11}$ and (b) $Yb_{13.5}La_{0.5}ZnSb_{11}$.

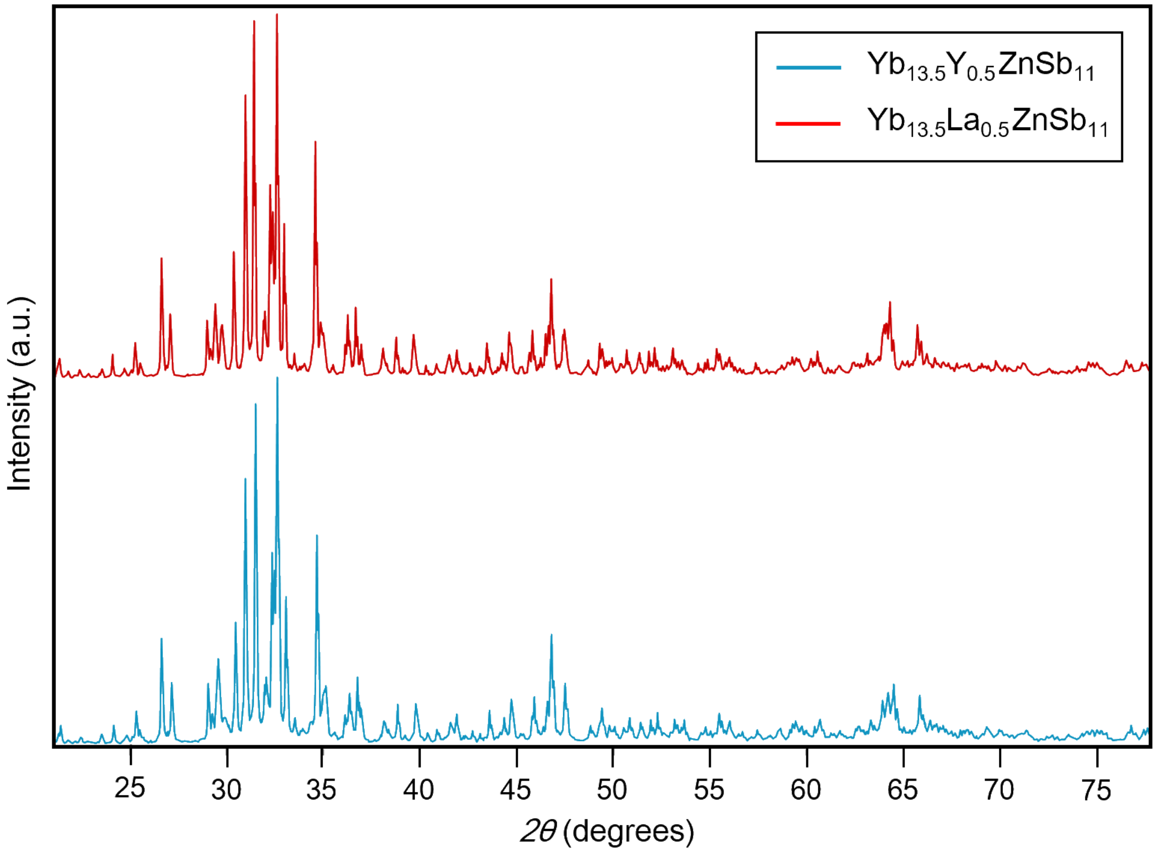

Figure S2. Powder X-Ray diffraction patterns from samples of $Yb_{13.5}Y_{0.5}ZnSb_{11}$ and $Yb_{13.5}La_{0.5}ZnSb_{11}$.

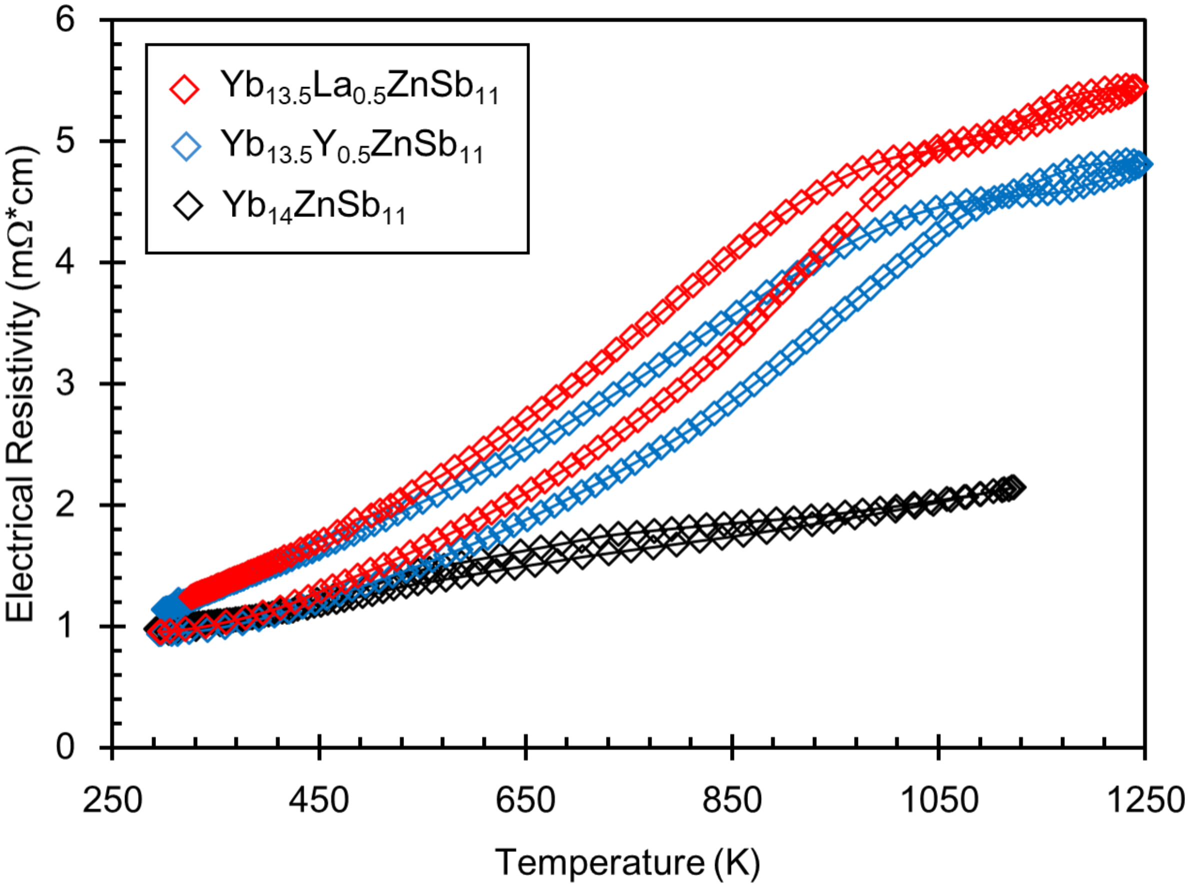

Figure S3. Electrical resistivity as a function of temperature for samples of $Yb_{13.5}Y_{0.5}ZnSb_{11}$, $Yb_{13.5}La_{0.5}ZnSb_{11}$ and $Yb_{14}ZnSb_{11}$ (data from Ref. 9).

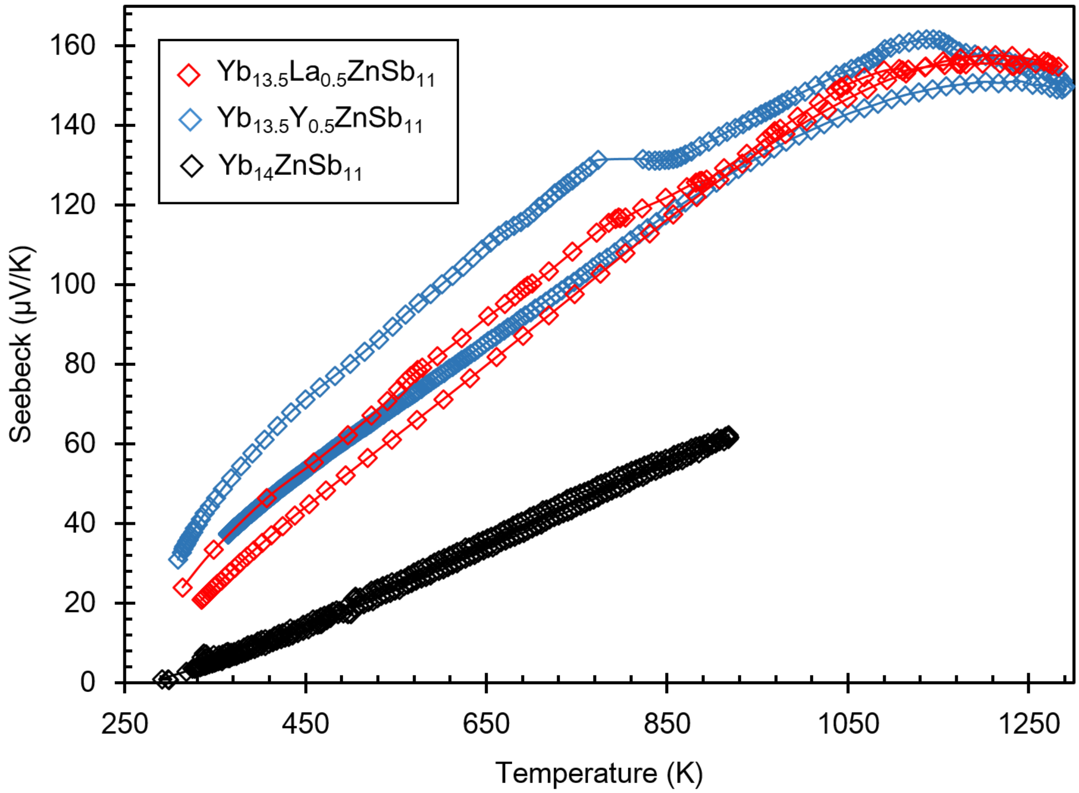

Figure S4. Seebeck as a function of temperature for samples of $Yb_{13.5}Y_{0.5}ZnSb_{11}$, $Yb_{13.5}La_{0.5}ZnSb_{11}$ and $Yb_{14}ZnSb_{11}$ (data from Ref 9).

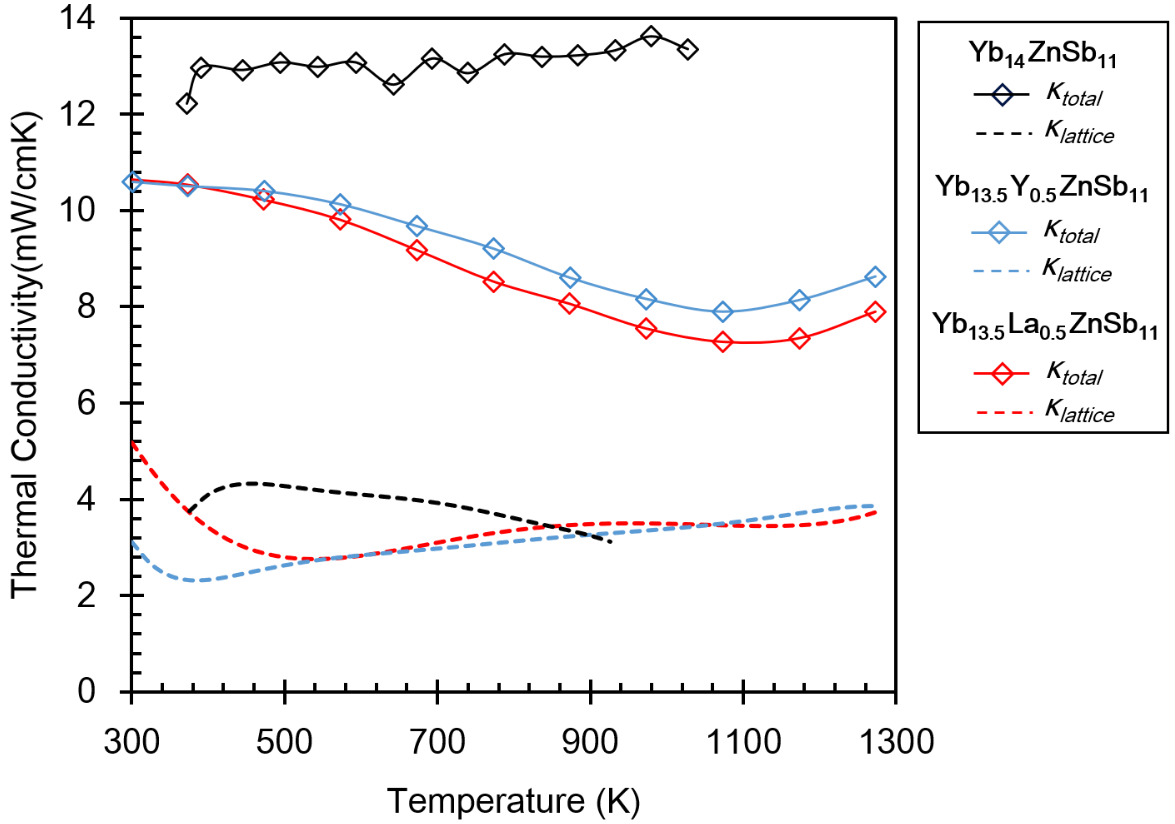

Figure S5. Total thermal conductivity and calculated lattice thermal conductivity as a function of temperature for samples of $Yb_{13.5}Y_{0.5}ZnSb_{11}$, $Yb_{13.5}La_{0.5}ZnSb_{11}$ and $Yb_{14}ZnSb_{11}$ (taken from Ref. 9).

9. Brown, S.R.; Toberer, E.S.; Ikeda, T.; Cox, C.A.; Gascoin, F.; Kauzlarich, S.M.; Snyder, G.J. Improved Thermoelectric Performance in $Yb_{14}Mn_{1-x}Zn_xSb_{11}$ by the Reduction of Spin-Disorder Scattering. *Chem. Mater.* **2008**, *20*, 3412–3419.
